# Swish and gargle saliva sampling is a patient-friendly and comparable alternative to nasopharyngeal swabs to detect SARS-CoV-2 in outpatient settings for adults and children

**DOI:** 10.1128/spectrum.01723-23

**Published:** 2023-10-20

**Authors:** Sandra Isabel, Justine Cohen-Silver, Hyejung Jung, Bridget Tam, Maya Lota, Marcia Sivilotti, Nancy Agbaje, Kevin L. Schwartz, Anne E. Wormsbecker, Larissa M. Matukas, Yan Chen

**Affiliations:** 1 Unity Health Toronto, Toronto, Ontario, Canada; 2 Department of Laboratory Medicine and Pathobiology, Temerty Faculty of Medicine, University of Toronto, Toronto, Ontario, Canada; 3 Department of Paediatrics, Temerty Faculty of Medicine, University of Toronto, Toronto, Ontario, Canada; 4 Li Ka Shing Knowledge Institute, Unity Health Toronto, Toronto, Ontario, Canada; 5 MAP Center for Urban Health Solutions, Toronto, Ontario, Canada; 6 Dalla Lana School of Public Health, University of Toronto, Toronto, Ontario, Canada; 7 Department of Medicine, Temerty Faculty of Medicine, University of Toronto, Toronto, Ontario, Canada; University of Mississippi Medical Center, Jackson, Mississippi, USA

**Keywords:** swish and gargle saliva, SARS-CoV-2 detection

## Abstract

Nasopharyngeal swabs (NPS) are considered the standard specimen for SARS-CoV-2 detection by PCR. We aimed to compare detection performance and patient experience of NPS with swish and gargle saliva (SGS) to determine its potential as an accurate, easy-to-collect, and comfortable alternative. We conducted a prospective study from March to May 2021 and recruited pediatric and adult outpatients seeking COVID-19 testing. Paired NPS and 5-mL SGS were collected from each participant during the same visit and tested in parallel using validated rRT-PCR assays. Variant of concern (VOC) analysis was performed for positive NPS specimens. The participant completed the surveys regarding their experience with the two collection methods. We included 238 participants in the SARS-CoV-2 detection performance analysis. Thirty-two participants tested positive for both NPS and SGS. In comparison to NPS, the sensitivity and specificity of SGS to detect SARS-CoV-2 were 94.1% (95%CI; 80.3%–99.3%) and 97.1% (95%CI; 93.7%–98.9%), respectively. Six participants tested positive only with SGS and only two with NPS. NPS is an imperfect gold standard. Thus, when compared to the COVID-19 case, that is, a patient with positive NPS and/or SGS, the sensitivity for NPS and SGS were 85.0% and 95.0%, respectively. VOC results (*n* = 26) revealed that 92% of the cases were alpha. From the 238 surveys, 89.9% of the participants described SGS collection as comfortable or very comfortable compared to 15.1% for NPS; 90.2% of the participants were likely or very likely to return for SGS collection compared to 59.3% for NPS. SGS is an alternative to NPS for SARS-CoV-2 detection in adult and pediatric outpatients as it is more patient-friendly while still maintaining comparable performance.

**IMPORTANCE:**

Widespread and frequent testing for COVID-19 was an important strategy to identify infected patients to isolate and control the spread of the disease during the pandemic. The nasopharyngeal swab (NPS) global supply chain and access to trained healthcare professionals for standard NPS collection were often compromised. Patient discomfort and limited access challenged health systems to reach large numbers for testing in adult and pediatric populations. Our study revealed that swish and gargle saliva (SGS) was comparable to NPS in detecting SARS-CoV-2 and more patient-friendly than NPS. Patients were more likely to repeat the test with SGS. SGS was amenable to self-collection instead of relying on skilled professionals. This comprehensive evaluation highlights the challenges of comparing the accuracy of new methods to imperfect gold standards and identifies additional patient-centric factors that should be considered when defining such standards. Thus, SGS is an advantageous alternative specimen collection for outpatient *en masse* testing.

## INTRODUCTION

The emergence of severe acute respiratory syndrome coronavirus 2 (SARS-CoV-2) resulted in the first outbreak of COVID-19 in December 2019 in Wuhan, China, followed by the World Health Organization (WHO) declaration of a pandemic in March 2020 (1). As of August 2023, the WHO reported more than 760 million confirmed COVID-19 cases worldwide (2). COVID-19 testing is considered an essential component of the strategy to contain and mitigate the COVID-19 pandemic. Similar to the detection of common respiratory viruses such as influenza, nasopharyngeal swab (NPS) collected by trained healthcare workers is the gold standard for SARS-CoV-2 detection with real-time reverse transcription polymerase chain reaction (rRT-PCR) (3, 4). A meta-analysis estimated the pooled sensitivity and pooled specificity of the NPS nucleic acid amplification test (NAAT) to be 84.8% and 98.9%, respectively (5). In Canada, as of 22 November 2022, more than 66 million SARS-CoV-2 rRT-PCR tests had been performed (6). However, testing capacity could be limited in areas lacking the necessary resources (e.g., skilled healthcare workers, personal protection equipment, swabs, and transport media). Additionally, patients’ experience with specimen collection could impact testing uptake as both adults and children are subjected to repetitive testing for a variety of indications such as COVID-19 symptoms or exposure and screening circumstances such as international travel and visits to long-term care facilities. Because the standard NPS is uncomfortable and can even be physically traumatic and distressing, particularly for children (7), alternative specimen collections should be assessed.

Saliva collection is a less invasive method of specimen collection compared to NPS. Different saliva collection approaches have been studied including direct saliva into an empty tube, direct saliva into a buffer media, and swish and gargle saliva admixed with spring water or normal saline into an empty tube (5, 8 – 10). Growing evidence shows the sensitivity of saliva in SARS-CoV-2 detection by rRT-PCR compared to NPS (5, 8 – 10) with one meta-analysis estimating saliva NAAT pooled sensitivity of 83.2% and specificity of 99.2% (5). Furthermore, Utama et al. reported that 97.1% of the patients recruited preferred self-collected gargle specimens instead of nasopharyngeal and oropharyngeal swabs for COVID-19 testing (11). However, challenges remain despite the implementation of COVID-19 testing using saliva specimens in several countries globally (10).

Given the potential discomfort of NPS, the need for frequent testing during the pandemic, and the promising sensitivity of saliva specimens, we aimed to identify an alternative specimen collection to NPS for COVID-19 testing that is convenient, user-friendly, comfortable, and noninvasive without compromising sensitivity and specificity in SARS-CoV-2 detection by rRT-PCR. We prospectively compare SARS-CoV-2 detection performance with swish and gargle saliva (SGS) and current NPS and include a survey to capture and analyze patient experience.

## MATERIALS AND METHODS

### Patient population, study design, and ethics

This is a prospective study conducted at Unity Health Toronto from 18 March to 12 May 2021, in two outpatient COVID-19 assessment centers and one pediatric clinic (CIBC Just for Kids Clinic, Toronto, Canada). The patients came for COVID-19 testing due to consistent symptoms or a history of exposure to known COVID-19 cases. The patients recruited were asked to provide first an NPS as the standard of care for SARS-CoV-2 rRT-PCR testing and then an additional voluntary SGS specimen after informed consent was obtained. Both children and adults were recruited without age limitation as long as they were able to provide an SGS specimen paired with the standard of care NPS. The participants or their parents were asked to complete a survey on their experience after specimen collection. We also included in the survey analysis answers of the participants whose specimens were excluded from the SARS-CoV-2 rRT-PCR testing analysis.

### Sample collection and rRT-PCR testing

An NPS in Roche Cobas PCR media (Roche Diagnostics, IN, USA) was collected for SARS-CoV-2 detection as the standard of care (12). The participants were also asked to self-collect an SGS sample after the healthcare worker collected the NPS. To collect the SGS, the participants were instructed to put 5 mL of normal saline in the mouth, swish for 5 s, gargle for 5 s, and repeat these two steps twice before spiting the saline in a collection tube through a well-fitted disposable plastic funnel or a paper straw (13). Following collection, all SGS and NPS were stored and transported at 4°C and then tested within 24 h from collection in compliance with standard laboratory procedures for COVID-19 testing in Ontario, Canada. Clinical information collected from the accompanying requisition included, but was not limited to, symptoms (if any), date of symptom onset, and date of exposure. Both NPS and SGS from a participant were excluded from the PCR analysis if either specimen was unlabeled, of insufficient volume (<1 mL), or leaking. All specimens were heat-inactivated for 40 min in an incubator at 65°C once they were received in the Molecular Diagnostic Laboratory at St. Michael’s Hospital, Unity Health Toronto (Toronto, Canada). After heat inactivation, the paired NPS and SGS specimens were tested together on one of three validated high-throughput platforms for SARS-CoV-2 detection. The platform was randomly chosen based on instrument availability. The three SARS-CoV-2 testing platforms and assays are listed below, and all included extraction and amplification exogenous internal controls used as per our clinically validated standard operative procedures and the manufacturers’ instructions. Each rRT-PCR reaction was analyzed to ensure that the exogenous internal control met pre-defined stringent parameters prior to reporting.

1. AltoStar Automation System AM16 and Bio-Rad CFX qPCR system: Realstar SARS-CoV-2 assay by Altona Diagnostics detecting S and N gene (Altona Diagnostics GmbH, Hamburg, Germany)
2. Seegene STARTlet automated extractor and Bio-Rad CFX qPCR system: Allplex 2019-nCoV assay by Seegene detecting N, E, and RdRp gene (Seegene Inc., Seoul, South Korea)
3. PerkinElmer Pre-NAT fully automatic system and Applied Biosystems QuantStudio 5 Real-Time PCR system: PerkinElmer SARS-CoV-2 assay detecting N and ORF1ab genes (PerkinElmer, MA, USA)

Of the 238 participants analyzed for SARS-CoV-2 with NPS and SGS, the paired specimens from the 103 participants were tested on the AltoStar system, 80 on the Seegene STARTlet system, and 55 on the PerkinElmer Pre-NAT fully automatic system. A specimen was reported as detected if the PCR cycle threshold (Ct) value was ≤40 for any SARS-CoV-2 gene target. When SARS-CoV-2 was detected in NPS with PCR Ct of ≤30, the NPS was subjected to VOC testing with a lab-developed test that distinguishes the wild type from the alpha variant (14).

### Sample size and statistical analyses

We calculated the sample size assuming a sensitivity of 95% in detecting SARS-CoV-2 for SGS in comparison to NPS as the reference standard, a COVID-19 prevalence at 12%, and a dropout rate at 3%. With a sensitivity of 75%, a specificity of 90% as the lower limit of the 95%CI, and an 80% power, the minimum study sample sizes were 232 and 234 based on sensitivity and specificity, respectively, with at least 27 NPS positive for SARS-CoV-2 and 199 NPS negative for SARS-CoV-2 (15).

The sensitivity and specificity of SGS in detecting SARS-CoV-2 were calculated in comparison to NPS as the reference standard. The McNemar test was used to determine if there are differences in a dichotomous test result between NPS and SGS. The co-primary analysis was performed using a composite reference standard, which was defined as positive if at least one of the NPS and SGS was positive and as negative if neither NPS nor SGS was positive. The sensitivities of SGS and NPS in diagnosing COVID-19 cases were calculated, respectively, compared to the composite reference standard. The specificity in diagnosing COVID-19 cases is considered to be 100% for both NPS and SGS in the co-primary analysis as the composite reference standard is used. Cohen’s kappa measured the degree of agreement between SGS and NPS in the data on a scale from –1 to 1. Negative values are interpreted as no agreement; 0–0.20 as none to slight; 0.21–0.39 as minimal; 0.40–0.59 as weak; 0.60–0.79 as moderate; 0.80–0.90 as strong; and >0.90 as almost perfect agreement (16). All statistical analyses were performed using R Studio version 1.3.1093 (RStudio Inc., Boston, MA, USA) and SAS version 9.4 (SAS Institute Inc., Cary, NC, USA).

### Survey collection and analysis

The participants were interviewed using 10 survey questions. Two asked about activities that may interfere with SARS-CoV-2 detection in SGS. Eight questions addressed the ease of self-collecting SGS, level of comfort when providing NPS and SGS samples, and likelihood of seeking COVID-19 testing again with NPS or SGS when indicated. The survey questions were as follows:

1. In the last 30 min, did you (your child) eat, drink, smoke, vape, chew gum, and brush your teeth (circle all that apply)?
2. Did you (your child) apply any of the following today: lip balm, lip gloss, and lipstick?
3. The instructions were easy to follow.
4. I (my child) was able to collect the specimen with little help.
5. I (my child) found it easy to gargle the saltwater.
6. The flavor of the salt water was tolerable.
7. How comfortable was it to give the swish and gargle saliva?
8. How comfortable was it to give the NP swab?
9. If you (your child) needed to be tested again, what is the likelihood that you would return for a swish and gargle of saliva?
10. If you (your child) needed to be tested again, what is the likelihood that you would return for a nasopharyngeal swab?

For each survey question, data were presented as the percentage for each five-point Likert scale using bar graphs (Fig. 2). Partially completed surveys were included. The responses for each of the eight questions were compared between adults and children using the exact Wilcoxon rank-sum test. The Bonferroni correction (0.05/8 = 0.00625) was used to adjust for multiple testing such that the probability of significance will be considered significant if it is less than or equal to 0.625%.

## RESULTS

### Participant demographics and exclusions

**Fig 1:**
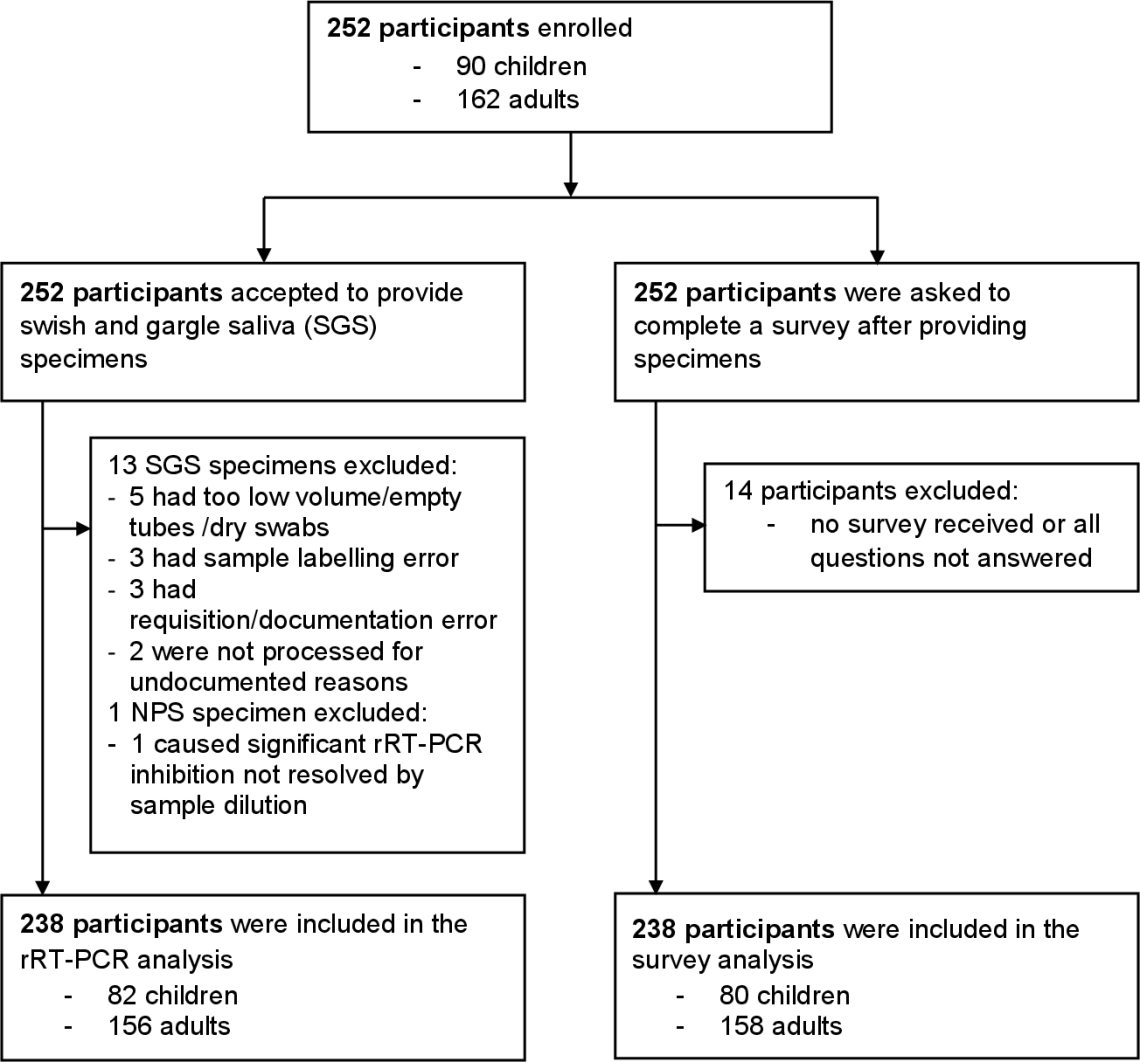
Cohort creation and exclusions for the two parts of this study: (i) performance characteristics and (ii) patient experience survey of SGS compared to NPS. We enrolled 252 participants, of whom 90 were children and 162 adults. We excluded 14 pairs of specimens from performance analysis, and coincidently 14 surveys were not answered or received. A total of 238 participants were included in the two parts of the study: rRT-PCR analysis and survey.

We enrolled 252 outpatients (90 children and 162 adults) in our prospective study (Fig. 1). Of them, 174 (69.0%) were symptomatic, 73 (29.0%) were asymptomatic with exposure to a known COVID-19 case, and five (2.0%) had no documented clinical information. The age of the participants ranged from 2 to 85 years, with 44.4% being female. We rejected 14 pairs of specimens for various reasons: insufficient volume of saliva, empty tubes, swabs of saliva, erroneous labels or documentation, and PCR inhibition by the NPS specimens. Among those, we recruited four patients with age from 2 to 3 years old. We rejected their specimens as SGS tubes were empty or saliva was collected with swabs. We could not find a documented reason for two additional rejected specimens. In addition, one NPS specimen inhibited the rRT-PCR, and we rejected the paired specimens for the study.

### Performance of SGS to detect SARS-CoV-2 using NPS as reference standard

We tested 238 paired SGS and NPS for SARS-CoV-2 with rRT-PCR; 32 participants tested positive for SARS-CoV-2 with both NPS and SGS specimens (Table 1). Eight participants had discrepant NPS and SGS results (Table 1), including two asymptomatic participants. Among these eight participants, two were symptomatic pediatric participants. Two participants tested positive only with NPS with a single gene target that was detected close to the lower limit of detection (i.e., Ct value > 35). These two participants were symptomatic and had specimens collected on day 6 or 7 from symptom onset. Six participants tested positive with SGS only with four of them detected close to the lower limit of detection. Two of these six were exposed asymptomatic participants and tested on day 2 or 7 since exposure, one participant had symptom onset on the day of collection, two had 1 day of symptoms, and one had 12 days of symptoms at the time of collection.

**TABLE 1:** SARS-CoV-2 rRT-PCR results of SGS compared to NPS as the reference standard

All participants	NPS		
SGS	Positive	Negative	Total
Positive	32	6	38
Negative	2	198	200
Total	34	204	238

When NPS was used as the reference standard, the SGS sensitivity and specificity were 94.1% (95%CI; 80.3%–99.3%) and 97.1% (95%CI; 93.7%–98.9%), respectively (Table 2). There was no statistical evidence of disagreement between NPS and SGS using the McNemar test (*P* = 0.16), and Cohen’s kappa agreement between SGS and NPS was 0.87 (95%CI; 0.78–0.96) indicating a strong agreement (16).

**TABLE 2:** Performance of SGS in different patient groups compared to NPS

All participants or subgroups (number of participants)	Sensitivity % (95% CI)	Specificity % (95% CI)
All participants (*n* = 238)	94.1 (80.3–99.3)	97.1 (93.7–98.9)
Symptomatic (*n* = 165)	93.3 (77.9–99.2)	97 (92.6–99.2)
Asymptomatic (*n* = 71)	100 (39.8–100)	97 (89.6–99.6)
Children (*n* = 82)	92.9 (66.1–99.8)	98.5 (92.1–100)
Adults (*n* = 156)	95 (75.1–99.9)	96.3 (91.6–98.8)

A total of 165 participants presented symptoms consistent with COVID-19. For this group, the SGS sensitivity and specificity were 93.3% (95%CI; 77.9%–99.2%) and 97% (95%CI; 92.6%–99.2%), respectively. Seventy-one participants were asymptomatic and were tested due to exposure to a known COVID-19 case. The SGS sensitivity and specificity for asymptomatic participants were 100% (95%CI; 39.8%–100%) and 97% (95%CI; 89.6%–99.6%), respectively. The clinical status (symptomatic/asymptomatic) was unknown for two participants; both resulted in concordant SARS-CoV-2 not detected.

We recruited 90 pediatric (<18 years old) participants and included 82 in the rRT-PCR performance analysis; the sensitivity and specificity were 92.9% (95%CI; 66.1%–99.8%) and 98.5% (95%CI; 92.1%–100%), respectively. We enrolled 162 adults (≥18 years old) and included 156 in the rRT-PCR performance analysis; the SGS sensitivity and specificity were 95% (95%CI; 75.1%–99.9%) and 96.3% (95%CI; 91.6%–98.8%), respectively.

### Performance of SGS and NPS in the detection of COVID-19 cases

We defined a COVID-19 case as a participant with either SGS or NPS, or both, positive for SARS-CoV-2. We reviewed the amplification curves of all gene targets for all positive specimens and determined they truly represented the detection of SARS-CoV-2. We calculated the sensitivity by comparing SGS and NPS to COVID-19 cases. The sensitivity for SGS was 95.0% (95%CI; 83.1%–99.4%) and for NPS was 85.0% (95%CI; 70.2%–94.3%) (Table 3). The specificity for SGS and NPS by definition was 100%.

**TABLE 3:** Performance characteristics of SGS and NPS in detecting COVID-19 cases^*a*^

All participants or subgroups (number of participants)	Specimen type analyzed	No. of positives	Sensitivity % (95% CI)	Specificity %
All participants (*n* = 238)	SGS	38	95.0 (83.1–99.4)	100.0
	NPS	34	85.0 (70.2–94.3)	100.0
Symptomatic (*n* = 165)	SGS	32	94.1 (80.3–99.3)	100.0
	NPS	30	88.2 (72.6–96.7)	100.0
Asymptomatic (*n* = 71)	SGS	6	100.0 (54.1–100.0)	100.0
	NPS	4	66.7 (22.3–95.7)	100.0
Adults (*n* = 156)	SGS	24	96.0 (79.7–99.9)	100.0
	NPS	20	80.0 (59.3–93.2)	100.0
Children (*n* = 82)	SGS	14	93.3 (68.1–99.8)	100.0
	NPS	14	93.3 (68.1–99.8)	100.0

^*a*^ Defined as a participant with either NPS or SGS or both positive for SARS-CoV-2.

We further examined the sensitivity of SGS and NPS to diagnose COVID-19 in different subgroups. The sensitivity in the symptomatic group was 94.1% (95%CI; 80.3%–99.3%) for SGS and 88.2% (95%CI; 72.6%–96.7%) for NPS. The sensitivity in the asymptomatic group was 100.0% (95%CI; 54.1%–100%) for SGS and 66.7% (95%CI; 22.3%–95.7%) for NPS. The sensitivity was 96.0% (95%CI; 79.7%–99.9%) for SGS and 80.0% (95%CI; 59.3%–93.2%) for NPS in adult participants and 93.3% (95%CI; 68.1%–99.8%) for both SGS and NPS in pediatric participants (Table 3).

### Variant of concern (VOC) testing in NPS positive for SARS-CoV-2

We conducted this study (18 March to 12 May 2021) when the SARS-CoV-2 alpha variant was predominant in Ontario, Canada (17). VOC results were available for 26 (of 34) NPS positive for SARS-CoV-2: 24 alpha (B.1.1.7), one gamma (P.1), and one wild type.

### Evaluation of factors affecting SARS-CoV-2 detection in SGS specimens

Many jurisdictions that have implemented SARS-CoV-2 PCR testing on saliva require no eating, drinking, or smoking within a certain time (e.g., 30 min) before saliva collection. We included participants even if they had done one or more of those activities in the 30 min prior to specimen collection. We requested the participants to report in the survey if they drank, ate, smoked, chewed gum, or brushed their teeth within 30 min before the SGS sample collection. Sixty-four participants reported at least one of these activities. Five of them tested positive for SARS-CoV-2. Four of those had paired SGS and NPS positive results, while one tested positive with SGS and negative with NPS. All other 59 participants had concordant negative results with SGS and NPS.

We also asked the participants to report if they were wearing lip balm, lip gloss, or lipstick. We had 30 participants who answered yes to this question; two had concordant SGS- and NPS-positive results, and the remaining 28 participants had concordant negative results. All specimens passed the pre-defined internal control parameters demonstrating no interference and result validity as true negatives.

### Patient experience survey

**Fig 2:**
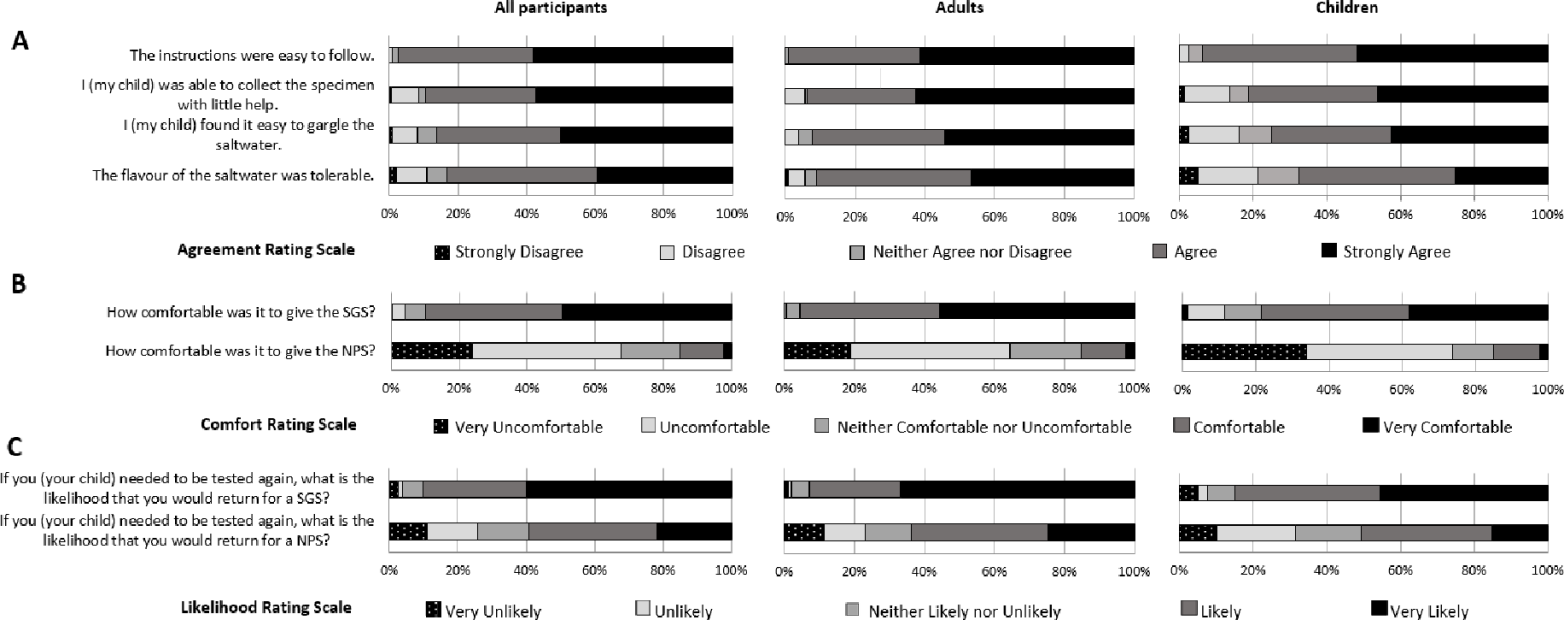
Patient experience survey: (A) regarding the agreement on the usability of the sample method, (B) regarding comfort with sampling), and (C) regarding the likelihood of using this sample method again.

We excluded 14 of the 252 participants enrolled (Fig. 1) from the survey analyses for two reasons: they did not answer any questions in the survey, or we did not receive a survey with the specimens (Fig. 1). We analyzed 238 surveys (80 children and 158 adults) and received answers from 235 to 238 (≥98.8%) participants for each question. A high proportion of the participants (89.9%; 213/237) reported that the SGS collection is comfortable or very comfortable while only 15.1% (36/238) for NPS. Although our survey results revealed that both adult and pediatric participants favored SGS collection methods, we found a higher portion of adult participants (95.6%; 151/158) compared to the pediatric group (78.5%; 62/79) that deemed the SGS collection method as comfortable or very comfortable (*P* = 0.0005). There is no significant difference noted in the proportion of adults and pediatric participants who considered NPS comfortable or very comfortable (Fig. 2) A higher proportion of the adult participants responded that they were able to collect an SGS sample with minimal help (93.7%; 148/158) when compared to children (81.2%; 65/80) (*P* = 0.005). The adult participants also expressed more tolerance to salty taste; 91.1% (144/158) of the adults agreed or strongly agreed compared to only 67.5% (54/80) of the children (*P* < 0.0001). Importantly, the perceived likelihood (likely or very likely) of returning for a test if needed was higher for the SGS collection method (90.3%; 213/236) compared to the NPS method (59.3%; 140/236). A higher proportion of adult participants are likely or very likely to return for a test if the SGS (93.0%; 146/157) collection is offered compared to children (84.8%; 67/79) (*P* = 0.001).

## DISCUSSION

We compared SGS with standard NPS to detect SARS-CoV-2 by rRT-PCR in both adult and pediatric outpatients. We included 238 participants (156 adults, 82 children) in the final performance analysis of SGS with 69.0% as symptomatic participants. When assigning NPS as the reference, SGS provides excellent sensitivity and specificity (94.1% and 97.1%) for SARS-CoV-2 detection. SGS also consistently exhibits excellent sensitivity (92.9% to 100%) and specificity (96.3% to 98.5%) in subgroup analysis on children, adults, and symptomatic and asymptomatic participants. When compared to clinical diagnosis of COVID-19 cases, defined as participants with either or both SGS and NPS positive specimens, SGS and NPS provide a sensitivity of 95.0% and 85.0%, respectively. Similar results were also observed in our subgroup analyses. From 238 surveys, the vast majority of participants described SGS collection as comfortable or very comfortable (89.9%), whereas a small proportion did for NPS (15.1%). In addition, the vast majority of the participants (90.3%) rated themselves as likely or very likely to return for SGS compared to more than half (59.3%) for NPS.

Studies showed that SARS-CoV-2 nucleic acids were present in saliva early in the pandemic (18). A later study investigating the reasons leading to smell and taste changes and oral lesions in patients with COVID-19 showed that SARS-CoV-2 replicates in the mouth’s oral mucosa and glands (19). Various forms of saliva specimen collections have been studied: direct saliva into an empty tube; direct saliva into a buffer-containing tube; and swishing and gargling of normal saline or spring water (5, 8). These studies reported a range of SARS-CoV-2 detection sensitivities for saliva specimens compared to NPS. The variation in ways of saliva collection is an important factor that can contribute to a wide range of sensitivities. Despite various forms of saliva studied for the detection of SARS-CoV-2 by rRT-PCR, Butler-Laporte et al.’s (5) meta-analysis showed that saliva and NPS had a similar pooled sensitivity of 83.2% (95% credible interval: 74.7%–91.4%) and 84.8% (95% credible interval: 98.2%–99.8%), respectively (5).

Goldfarb et al. (9) evaluated SGS, collected the same way as in our study, for diagnosis of COVID-19 in children (9). Their calculated sensitivity was 98% for an outpatient pediatric population (*n* = 40). Of these 40 patients, 36 were known positive cases initially confirmed by NPS and retested with SGS with a median time of 3 days from infection confirmation to retesting. The remaining 14 patients (household contacts/symptomatic children) provided both NPS and SGS at the time of visit, and at least one specimen was positive for SARS-CoV-2 (9). In our study, the specimen collection was performed on the same visit in children and adults. Our findings were consistent with those of Goldfarb et al. and added statistical evidence as a result of a larger study size and a prospective study design. Goldfarb et al. conducted their study in BC Children’s Hospital between May and September 2020 when wild-type SARS-CoV-2 was in circulation in Canada (9), while our study was done during a wave of the pandemic when the alpha variant was the predominant VOC.

NPS has been accepted as the standard specimen for SARS-CoV-2 detection using rRT-PCR since historically NPS was used to detect other respiratory viruses, such as influenza virus A (3). However, there has been no clearly defined gold standard specimen for SARS-CoV-2 detection. Gao et al. evaluated SARS-CoV-2 detection by rRT-PCR in both NPS and bronchial alveolar lavage (BAL) specimens in ICU patients with respiratory failure. They observed 14 cases with discrepant results; five patients tested positive with only NPS, while SARS-CoV-2 was detected only in BAL for nine patients (4). To overcome the limitation of comparing SGS to an imperfect gold standard, NPS, we evaluated the performance of SGS and NPS in the detection of COVID-19 cases. We defined a COVID-19 case as a participant with either SGS or NPS, or both, positive for SARS-CoV-2. Both NPS and SGS were used to estimate the true disease status of a participant. The use of a composite reference, such as the COVID-19 case definition used in our study, should be considered to avoid bias as a result of an imperfect gold standard.

We had a small number of discrepant results between NPS and SGS for SARS-CoV-2 detection. Six out of the eight discrepancies showed positive SGS and negative NPS, while only two showed the opposite pattern. These SGS-only positives were predominantly detected within the first day of symptom onset or when they were asymptomatic. While our study was not powered nor designed to specifically address sensitivity over time, it is interesting to note that the discrepant results where SGS was positive but NPS was negative appear to be consistent with the SARS-CoV-2 human challenge study in the UK. They showed that infection with the omicron variant started in the throat and then viral particles were more abundant in the nose than the throat after 5 days from inoculation (20). This phenomenon of virus detection in different anatomical compartments of the upper respiratory tract was also studied by Congrave-Wilson et al. (21) who recruited patients with confirmed household exposure from Children’s Hospital Los Angeles and nearby community testing centers to look at changes in SARS-CoV-2 detection by rRT-PCR over the course of infection (21). The participants provided paired nasopharyngeal and saliva samples every 3 to 7 days for up to 4 weeks or until two negative PCR test results with NPS were obtained. Their group found that SARS-CoV-2 detection in saliva was highest during the first week of infection and decreased each subsequent week when compared to NPS. We hypothesize that the findings from both studies provide supporting evidence that SGS may have advantages in detecting the early stage of infection. This may influence the specimen collection type based on days from symptom onset. Further studies to address this specifically are needed.

While NPS collection for SARS-CoV-2 rRT-PCR is the standard of care for diagnosis of COVID-19, it requires trained health professionals and importantly can cause discomfort. Patients, particularly children, often are reluctant to seek COVID-19 testing again after NPS collection. Previous studies used sponge-based kits to collect saliva in the 6- to 8-year-old children and expectoration of 1 mL of saliva in a tube in children aged 9 years and above (7). That study assessed the pain associated with NPS and saliva collection (*n* = 48), and most rated the pain at 4/10 or higher for NPS versus 0/10 for saliva. For SGS, another study assessed the mean acceptability at 4.95 on a five-point Likert scale (9). Our survey focused on the patient experience with SGS for SARS-CoV-2 detection and identified this method to be a patient-friendly, self-collectable specimen collection. In our study, children as young as 4-year-old were able to provide SGS with help from their parents. Children aged 4–9 years old (27 enrolled) all provided sufficient volume of saliva. A prior survey of children assessed the pain associated with NPS and saliva collection. Our survey results identified that the vast majority of adult and pediatric participants considered SGS as easy and comfortable for self-collection. The improved patient experience with SGS most likely explained why 30.9% more participants would agree to test again if needed when SGS collection is offered. This preference was observed in both pediatric and adult participants, providing evidence that SGS may increase the uptake of COVID-19 testing among all patients. We found a statistically significant difference in the experience with SGS between adults and children. More adults tolerated the salty taste of saline and found SGS easy to collect compared to children. It is thus important to evaluate the experience of adults and children separately as their perceptions and understanding can be different. This should be taken into account by clinicians ordering the testing and by clinical microbiology laboratory staff implementing the methods.

We believe that SGS collection for SARS-CoV-2 detection has advantages during the current pandemic predominated by the omicron strain. The omicron strain is highly transmissible, and increasing testing uptake would be an effective strategy for identifying cases in order to contain outbreaks. Ansil’s findings suggested that increased test uptake was more effective than increased test sensitivity in the lowering probability of COVID-19 outbreaks (22). When omicron became predominant, SARS-CoV-2 rRT-PCR testing on self-collected SGS specimens among healthcare workers in our hospital network and shelters was strategically implemented to increase test uptake as a way to curb transmission of omicron and to minimize nosocomial COVID-19 outbreaks.

As limitations, our study was neither powered nor designed to address the performance differences in SARS-CoV-2 detection with SGS between different subgroups. We were unable to recruit enough children with COVID-19 to attain statistical power for this subgroup as schools were closed by a provincial stay-at-home order (7 April 2021) during our study period (23). The youngest participants able to provide SGS specimens with the help of the parent were 4 years old in this study. We recognize that the SGS could still be difficult to collect for young children. More studies are warranted to focus on the youngest population without the oropharyngeal motor skills needed for SGS for improved testing strategy.

We tested paired specimens randomly on three different SARS-CoV-2 testing platforms. There was no repeat testing on discrepant pairs in our study protocol as this study was designed and conducted to mirror our testing procedure on clinical specimens. These discrepant results may have been due to one of the specimen types harboring viral loads at the lower limit of detection of the assay or less likely contamination of one of the specimen types, since stringent quality control and contamination monitoring protocols were in place. Our ethics approval did not include a detailed chart review to collect additional clinical information when discrepant results were found and confirm COVID-19 diagnosis with clinical information or repeated testing if available. We also evaluated in our study the factors such as drinking, eating, and smoking prior to SGS collection for their impact on SARS-CoV-2 detection. We had 64 patients who performed these activities; four patients were positive with both SGS and NPS, and only one was positive with SGS. We did not identify any issue with detection but a larger study size is needed to draw a definitive conclusion. In order to adhere to the infection control measures, the healthcare worker in the assessment center or clinic involved in this study verbally administered the survey. This could have influenced patient responses as the healthcare worker may have also collected the NPS. A small proportion of patients reported NPS as comfortable or very comfortable (only 15.1%) compared to 89.9% for SGS, which suggests this trend is likely representative of their experience and not influenced by the healthcare worker’s presence. Finally, our study was conducted when alpha VOC was predominant, while Omicron VOC is now predominant and other VOCs will likely arise.

### Conclusion

We showed that SGS is equivalent to NPS in sensitivity and specificity to detect SARS-CoV-2 with rRT-PCR in outpatients. Given that SGS is self-collectible and a more comfortable collection method, this could lead to a higher return for testing than NPS, directing it to be the preferred standard for collection. Indeed, when defining gold standards, we propose that accuracy should no longer be the sole performance characteristic evaluated as patient experience influences COVID-19 testing uptake. SGS should be recommended as an equivalent alternative to NPS in outpatients, particularly when a self-collection approach would provide advantages in settings such as schools, long-term care facilities, shelters, and (essential) worker screening.

## SUPPLEMENTAL MATERIAL

Table S1 spectrum.01723-23-s0001.docx Summary of SGS and NPS specimens with SARS-CoV-2 detected.

ASM does not own the copyrights to Supplemental Material that may be linked to, or accessed through, an article. The authors have granted ASM a non-exclusive, world-wide license to publish the Supplemental Material files. Please contact the corresponding author directly for reuse.
