# Supplementary material for: Swish and gargle saliva sampling is a patient-friendly and comparable alternative to nasopharyngeal swabs to detect SARS-CoV-2 in outpatient settings for adults and children
Source: Microbiol Spectr. 2023 Oct 20;11(6):e01723-23. doi: 10.1128/spectrum.01723-23 (PMC10714816; doi:10.1128/spectrum.01723-23)
Supplement: Table S1 — Summary of SGS and NPS specimens with SARS-CoV-2 detected. [file spectrum.01723-23-s0001.docx]

**Supplementary table 1. Summary of SGS and NPS specimens with SARS-CoV-2 detected**

|  | **Instrument** | **Ct Values of Gene Targets** | | **Presence of Symptoms** | **No. of Days since Symptom Onset** | **No. of Days since Exposure** | **VOC Type** |
| --- | --- | --- | --- | --- | --- | --- | --- |
|  |  | **SGS** | **NPS** |  |  |  |  |
| 1 | AltoStar® | S: 20.16 | S: 16.18 | Symptomatic | 3 | NA | B1.1.7 |
|  |  | E: 19.37 | E: 15.1 |  |  |  |  |
| 2 | AltoStar® | S: 30.85 | S: 33.22 | Symptomatic | 6 | NA | NA |
|  |  | E: 30.68 | E: 33.25 |  |  |  |  |
| 3 | AltoStar® | S: 29.63 | S: 29.42 | Symptomatic | 0 | NA | NA |
|  |  | E: 28.44 | E: 28.9 |  |  |  |  |
| 4 | AltoStar® | S: 33.73 | S: 27.67 | Symptomatic | 6 | NA | B1.1.7 |
|  |  | E: 32.79 | E: 27.15 |  |  |  |  |
| 5 | AltoStar® | S: 32.06 | S: 33.29 | Symptomatic | 8 | NA | NA |
|  |  | E: 31.48 | E: 33.62 |  |  |  |  |
| 6 | AltoStar® | S: 23.01 | S: 16.24 | Symptomatic | NA | NA | B1.1.7 |
|  |  | E: 22.29 | E: 15.22 |  |  |  |  |
| 7 | AltoStar® | S: 23.88 | S: 19.85 | Symptomatic | 0 | NA | B1.1.7 |
|  |  | E: 23.09 | E: 18.27 |  |  |  |  |
| 8 | AltoStar® | S: 29.55 | S: 17.48 | Symptomatic | 4 | NA | B1.1.7 |
|  |  | E: 30.01 | E: 17.51 |  |  |  |  |
| 9 | AltoStar® | S: 18.03 | S: 20.74 | Symptomatic | 1 | NA | B1.1.7 |
|  |  | E: 17.42 | E: 20.33 |  |  |  |  |
| 10 | AltoStar® | S: 33.98 | S: 17.44 | Symptomatic | 4 | NA | B1.1.7 |
|  |  | E: 33.23 | E: 16.33 |  |  |  |  |
| 11 | AltoStar® | S: 20.42 | S: 25.46 | Symptomatic | 1 | NA | B1.1.7 |
|  |  | E: 20.73 | E: 24.91 |  |  |  |  |
| 12 | AltoStar® | S: 28.68 | S: 31.6 | Symptomatic | 1 | NA | NA |
|  |  | E: 29.15 | E: 32.28 |  |  |  |  |
| 13 | AltoStar® | S: UND | S:35.3 | Symptomatic | 7 | NA | NA |
|  |  | E: UND | E: UND |  |  |  |  |
| 14 | AltoStar® | S: 21.96 | S: 19.94 | Symptomatic | 3 | NA | P.1 |
|  |  | E: 22.02 | E: 20.95 |  |  |  |  |
| 15 | AltoStar® | S: 20.6 | S: 14.58 | Symptomatic | 2 | NA | Wild type |
|  |  | E: 19.8 | E: 13.24 |  |  |  |  |
| 16 | Seegene STARTLET | E: 19.17 | E: 15.48 | Symptomatic | 1 | NA | B1.1.7 |
|  |  | RdRp: 21.3 | RdRp: 17.63 |  |  |  |  |
|  |  | N: 21.26 | N: 17.49 |  |  |  |  |
| 17 | Seegene STARTLET | E: 35.17 | E: UND | Asymptomatic | NA | 2 | NA |
|  |  | RdRp: 35.39 | RdRp: UND |  |  |  |  |
|  |  | N: 34.41 | N: UND |  |  |  |  |
| 18 | Seegene STARTLET | E: 14.99 | E:25.1 | Symptomatic | 1 | NA | B1.1.7 |
|  |  | RdRp: 17.31 | RdRp: 27.19 |  |  |  |  |
|  |  | N: 16.51 | N: 26.99 |  |  |  |  |
| 19 | Seegene STARTLET | E: 28.09 | E: 20.08 | Symptomatic | 5 | NA | B1.1.7 |
|  |  | RdRp: 30.35 | RdRp: 22.29 |  |  |  |  |
|  |  | N: 29.67 | N: 21.2 |  |  |  |  |
| 20 | Seegene STARTLET | E: 29.95 | E: UND | Symptomatic | 1 | NA | NA |
|  |  | RdRp: 31.72 | RdRp: UND |  |  |  |  |
|  |  | N: 31.96 | N: UND |  |  |  |  |
| 21 | PerkinElmer® | N: 39.01 | N: 18.72 | Symptomatic | 4 | NA | B1.1.7 |
|  |  | ORF1ab: 37.86 | ORF1ab: 20.75 |  |  |  |  |
| 22 | PerkinElmer® | N: 22.62 | N: 21.79 | Symptomatic | 5 | NA | B1.1.7 |
|  |  | ORF1ab: 22.12 | ORF1ab: 23.67 |  |  |  |  |
| 23 | PerkinElmer® | N: 28.78 | N: UND | Symptomatic | 0 | NA | NA |
|  |  | ORF1ab: 27.4 | ORF1ab: UND |  |  |  |  |
| 24 | Seegene STARTLET | E: 26.53 | E: 22.85 | Symptomatic | NA | NA | B1.1.7 |
|  |  | RdRp: 28.74 | RdRp: 24.72 |  |  |  |  |
|  |  | N: 28.23 | N: 23.59 |  |  |  |  |
| 25 | AltoStar® | S: 19.53 | S: 15.95 | Symptomatic | NA | NA | B1.1.7 |
|  |  | E: 18.4 | E: 15.06 |  |  |  |  |
| 26 | Seegene STARTLET | E: UND | E: UND | Symptomatic | 12 | NA | NA |
|  |  | RdRp: UND | RdRp: UND |  |  |  |  |
|  |  | N: 38.99 | N: UND |  |  |  |  |
| 27 | Seegene STARTLET | E: 27.16 | E: 27.59 | Symptomatic | 7 | NA | B1.1.7 |
|  |  | RdRp: 30.18 | RdRp: 29.57 |  |  |  |  |
|  |  | N: 29.02 | N: 28.69 |  |  |  |  |
| 28 | PerkinElmer® | N: 35.86 | N: UND | Asymptomatic | NA | 7 | NA |
|  |  | ORF1ab: 34.19 | ORF1ab: UND |  |  |  |  |
| 29 | PerkinElmer® | N: UND | N: 38.38 | Symptomatic | 6 | NA | NA |
|  |  | ORF1ab: UND | ORF1ab: UND |  |  |  |  |
| 30 | AltoStar® | S: 25.07 | S: 18.1 | Symptomatic | NA | NA | B1.1.7 |
|  |  | E: 23.96 | E: 17.17 |  |  |  |  |
| 31 | AltoStar® | S: 20.98 | S: 19.65 | Asymptomatic | NA | NA | B1.1.7 |
|  |  | E: 20.61 | E: 19.29 |  |  |  |  |
| 32 | AltoStar® | S: 19.3 | S: 15.08 | Symptomatic | 2 | NA | B1.1.7 |
|  |  | E: 18.49 | E: 14.02 |  |  |  |  |
| 33 | AltoStar® | S: 19.04 | S: 14.38 | Symptomatic | 2 | NA | B1.1.7 |
|  |  | E: 18.16 | E: 13.27 |  |  |  |  |
| 34 | AltoStar® | S: 20.24 | S: 16.17 | Symptomatic | 2 | NA | B1.1.7 |
|  |  | E: 19.23 | E: 15.36 |  |  |  |  |
| 35 | AltoStar® | S: 22.31 | S: 17.2 | Symptomatic | 2 | NA | B1.1.7 |
|  |  | E: 21.31 | E: 16.11 |  |  |  |  |
| 36 | Seegene STARTLET | E: 34.52 | E: UND | Symptomatic | 1 | NA | NA |
|  |  | RdRp: UND | RdRp: UND |  |  |  |  |
|  |  | N: 36.6 | N: UND |  |  |  |  |
| 37 | AltoStar® | S: 22.59 | S: 23.6 | Symptomatic | 1 | NA | B1.1.7 |
|  |  | E: 22.85 | E: 23.54 |  |  |  |  |
| 38 | AltoStar® | S: 23.81 | S: 33.45 | Asymptomatic | NA | 2 | NA |
|  |  | E: 24.19 | E: 34.59 |  |  |  |  |
| 39 | AltoStar® | S: 20.39 | S: 32.58 | Asymptomatic | NA | 2 | NA |
|  |  | E: 20.7 | E: 32.15 |  |  |  |  |
| 40 | AltoStar® | S: 16.87 | S: 25.26 | Asymptomatic | NA | 2 | B1.1.7 |
|  |  | E: 17.26 | E: 24.72 |  |  |  |  |

**Legend.** UND: Undetermined
